# Supplementary material for: A Handy Flexible Micro-Thermocouple Using Low-Melting-Point Metal Alloys
Source: Sensors (Basel). 2019 Jan 14;19(2):314. doi: 10.3390/s19020314 (PMC6359204; doi:10.3390/s19020314)
Supplement: Supplementary file 1 [file sensors-19-00314-s001.zip › supplementary document.docx]

**Supplementary materials**

**Content**

[**1.** **Graphs of scanning electron microscope** 2](#_Toc533808190)

[**2.** **DSC results** 4](#_Toc533808191)

[**3.** **Heating/cooling cycle tests** 5](#_Toc533808192)

[**4. Before/after bending test** 7](#_Toc533808193)

1. **Graphs of scanning electron microscope**

Energy spectrum diagrams of alloy mixtures with different mixing ratio were listed as follows.

Figure 2.1 shows the energy spectrum diagrams of alloy mixtures with 40% EBiInSn. In these figures, Bi was not detected by SEM. It can be seen in Fig. 2.1(i) that there are a lot of mountain-like rigid structures. We believe these rigid structures are Bi-based alloy. The only reason that Bi was undetectable is that the surface of the Bi-based alloy was covered by a layer of Ga-based alloy. The effective detecting depth of SEM is 50 um. Therefore, there are too much Ga-based alloy and the thickness of Ga-based alloy layer is larger than 50 um. As shown in Figure 2.2~Figure 2.6, when more Bi-based alloy was added into the mixture, the Bi became detectable and more clear in the SEM images.

**
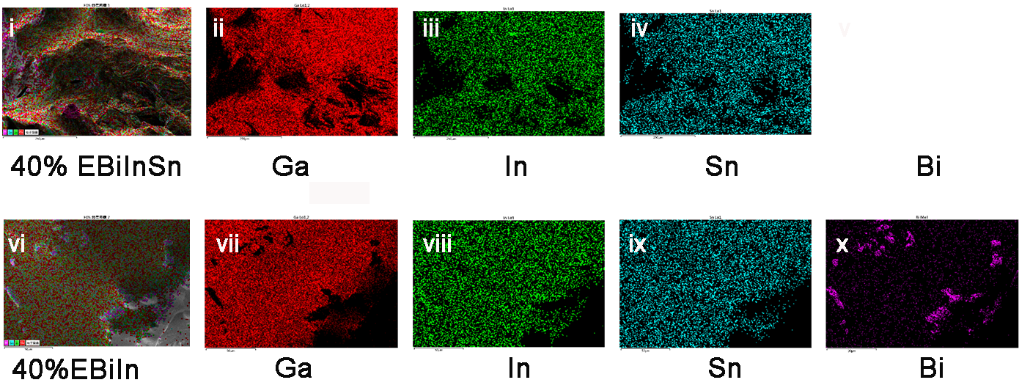
**

**Figure 2.1.** Energy spectrum diagrams of alloy mixtures with 40% mass ratio of Bismuth based alloys

**
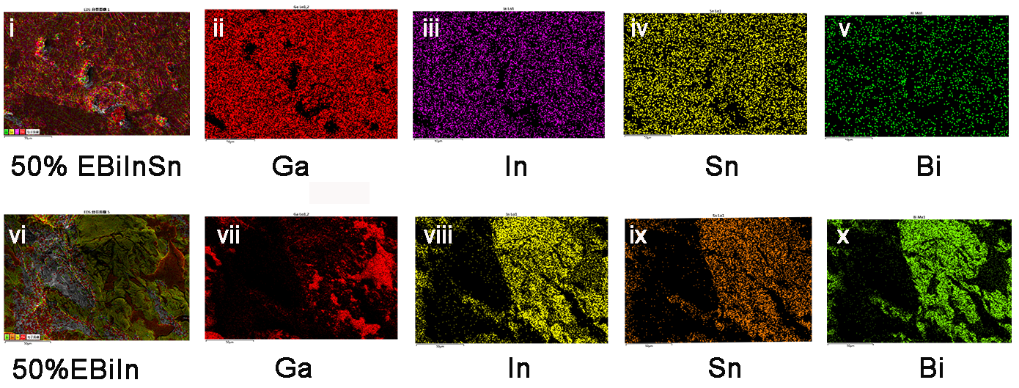
**

**Figure 2.2.** Energy spectrum diagrams of alloy mixtures with 50% mass ratio of Bismuth based alloys

**
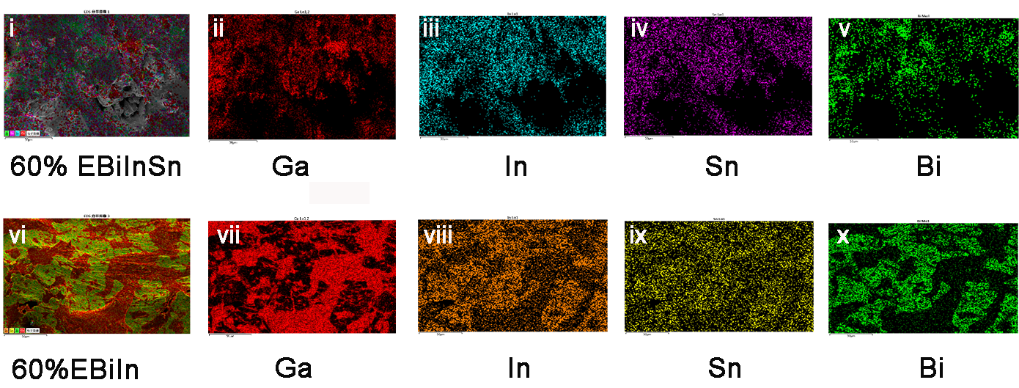
**

**Figure 2.3.** Energy spectrum diagrams of alloy mixtures with 60% mass ratio of Bismuth based alloys

**
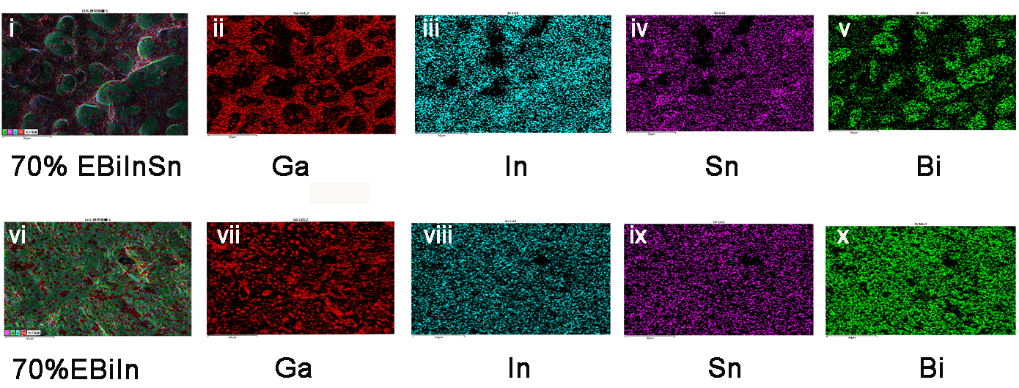
**

**Figure 2.4.** Energy spectrum diagrams of alloy mixtures with 70% mass ratio of Bismuth based alloys

**
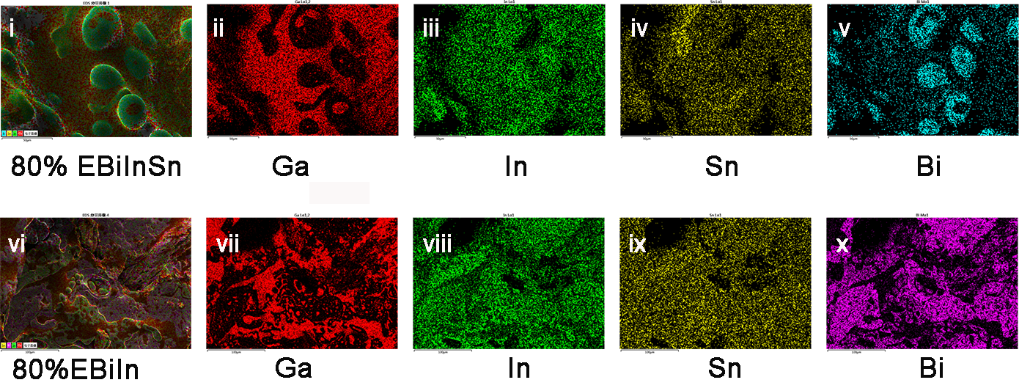
**

**Figure 2.5.** Energy spectrum diagrams of alloy mixtures with 80% mass ratio of Bismuth based alloys

**
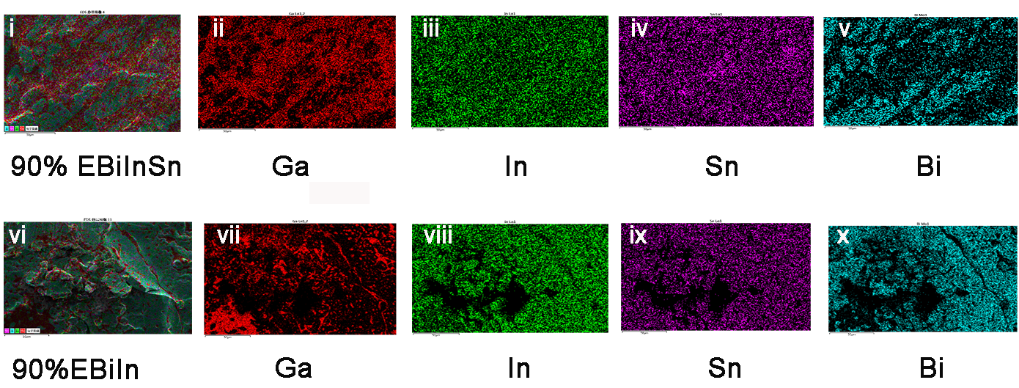
**

**Figure 2.6.** Energy spectrum diagrams of alloy mixtures with 90% mass ratio of Bismuth based alloys

1. **DSC results**

DSC graphs of alloy mixtures with different mass ratio of Bismuth based alloys are listed as follows.

In all these figures, they had four peaks. The first and fourth peaks represented the melting endothermic process and the solidification exothermic process of one metal alloy in the mixture, respectively, which showed that the melting point of this metal alloy was approximately 11 °C. This melting point coincides with the melting point of EGaInSn. Thus the liquid portion of the mixture was EGaInSn. Similarly, Figure. 2 (a-f) shows that he melting points of the second metal were around 42°C, which was lower than that of EBiInSn (60 °C). Figures 2 (g-l) show that the melting points of the second metal were around 75°C for EBiIn mixture. The melting point of this new metal was lower than that of EBiIn (109 °C). According to the SEM results, the Sn will transfer easily between the Ga-based alloy and Bi-based alloy. This Sn transfer phenomena may cause the new metals. According to the results, all the mixtures behave quite same.







(a) (b)







(c) (d)







(e) (f)







(g) (h)







(i) (j)







(k) (l)

**Figure 2.** DSC graphs of alloy mixtures with different mass ratio of Bi-based alloys

1. **Heating/cooling cycle tests**

All the chips were tested in the heating/cooling process for three cycles. The results are listed as follows.

Figures (a-f) show that the chips have good linearity between 25 °C and 55 °C in the heating/cooling cycle tests. When the temperature was higher than 55 °C. The change of the voltage was unstable. Figures (g-l) illustrated the similar phenomenon which the effective working range is from 25 °C to 83 °C. The data about 70% and 90% EBiIn was incomplete because the chip was damaged when it was heated above 80 °C.



**

**

（a）

**



**

（b）

**



**

（c）

**



**

（d）

**



**

（e）

**



**

（f）

**



**

（g）

**



**

（h）

**

**



（i）





（j）







（k）





（l）

**Figure 3.** Plots of Heating/cooling process results with different mass ratio of Bismuth based alloys.

1. **Test before/after bending test**

Figure 4 show that bending test results of the performance of mixed alloys thermocouples. The Seebeck coefficients are almost same before/after the bending test in the working range.

**



**

1. (b)

**Figure 4.** Plots of bending test results with different mass ratio of Bismuth based alloys.
